# IgG4-related retroperitoneal fibrosis overlapping with primary biliary cirrhosis and primary Sjögren's syndrome: A case report

**DOI:** 10.1097/MD.0000000000011303

**Published:** 2018-06-29

**Authors:** Xuan Huang, Bin Lu, Meng Li, Yihong Fan, Lu Zhang

**Affiliations:** Department of Gastroenterology, First Affiliated Hospital of Zhejiang Chinese Medical University, Zhejiang, China.

**Keywords:** immunoglobulin G4-related retroperitoneal fibrosis, primary biliary cirrhosis, primary Sjögren's syndrome

## Abstract

**Rationale:**

IgG4-related disease (IgG4-RD) is a chronic fibro-inflammatory disorder which is characterized by elevated levels of serum IgG4 and infiltration of IgG4-bearing plasma cells in the involved organs. Primary biliary cirrhosis (PBC) and Primary Sjögren's syndrome (pSS) are both distinct from IgG4-related disease. We herein describe a Chinese patient with IgG4-related RPF overlapping with PBC and pSS.

**Patient concerns:**

We report a case of 69-year-old male with recurrent lower abdominal pain for 10 months. Laboratory data showed elevated erythrocyte sedimentation rate and hepatobiliary enzymes, renal dysfunction, high titers of antinuclear antibody, anti-SS-A antibody and anti-mitochondrial type 2, high immunoglobulin (Ig) G levels and elevated serum IgG4 (9 g/L). Contrast-enhanced computed tomography and magnetic resonance imaging were suggestive of retroperitoneal fibrosis and unilateral ureteral occlusion. Immunohistochemical staining for IgG4 did not demonstrate infiltration of IgG4-positive plasma cells in the retroperitoneal mass, but revealed significant infiltration of lymphocytoplasma cells as well as fibrosis and fibrin accumulation.

**Diagnoses:**

The patient was diagnosed with IgG4-related retroperitoneal fibrosis based on the International Consensus Diagnostic Criteria. He was also diagnosed with primary biliary cirrhosis and primary Sjögren's syndrome.

**Interventions:**

250 mg ursodeoxycholic acid was administered twice daily, and prednisolone was initiated at a dose of 40 mg/day and then tapered to 25 mg after 45 days.

**Outcomes:**

The size of the retroperitoneal soft tissue mass gradually reduced and the abnormal laboratory parameters were restored to normal.

**Lessons:**

This rare clinical condition has seldom been reported in the literature, which suggests that common immunogenetic factors may be involved in the development of IgG-related RPF, PBC and pSS.

## 1 Introduction

Retroperitoneal fibrosis (RPF) is an uncommon disorder of unknown etiology that encompasses several different pathophysiologic entities and is characterized by the development of extensive fibrosis throughout the retroperitoneum. Due to the wide availability of sensitive diagnostic methods, the estimated annual incidence of RPF has increased to 1.3/100,000 inhabitants.^[1]^

IgG4-related disease (IgG4-RD) is a chronic fibro-inflammatory disorder which is characterized by elevated levels of serum IgG4 and infiltration of IgG4-bearing plasma cells in the involved organs. The condition essentially affects any organ in the body, including the pancreas, kidneys, lungs, lacrimal glands, salivary glands and retroperitoneal cavity. Since Hamano first reported the complications of IgG4-associated RPF, IgG4-RD has been listed as one of the causes of RPF.^[2]^

Salivary glands and bile ducts are frequently involved in multi-organ IgG4-RD. Involvement of the former is referred to as Mikulicz's disease, which is associated with elevated serum IgG4 levels and prominent infiltration of IgG4-positive plasmacytes.^[3]^ Involvement of the latter is referred to as IgG4-related sclerosing cholangitis (IgG4-SC), which can present typical imaging features of a thickened bile duct wall with segmental or diffuse biliary strictures, elevated serum IgG4 levels and classic histological features.^[4]^ Although primary biliary cirrhosis (PBC) and primary Sjögren's syndrome (pSS) are both well-defined autoimmune diseases, they are both distinct from IgG4-RDs such as Mikulicz's disease and IgG4-SC.

In this study, we describe a Chinese patient with IgG4-related RPF overlapping with PBC and pSS. We obtained informed consent from the patient for reporting this case. This rare clinical condition has seldom been reported in the literature.

## 2 Case report

A 69-year-old male farmer presented to our hospital for evaluation of mild left lower abdominal pain. The onset of symptoms occurred ten months prior to his admission to our hospital. The patient was previously admitted to another provincial hospital and misdiagnosed with abdominal aortic dissection. His symptoms were not improved following the administration of oral antibiotics and proton pump inhibitors.

Physical examination was unremarkable except for upper right abdomen and periumbilical mild tenderness. Complete blood counts revealed mild anemia (Hb 10.2 g/dL) with normal leukocyte and platelet counts. Biochemistry showed elevated levels of γ-glutamyltransferase (103.0 U/L, range: 7–45 U/L), alkaline phosphatase (144 U/L, range: 50–135 U/L), renal dysfunction [serum creatinine (113.99 μmol/L, range: 45–84 μmol/L)], and elevated levels of serum amylase (138 U/L, range: 15–125 U/L). The erythrocyte sedimentation rate was elevated at 84 mm/h (range: 0–20 mm/h). Total serum IgG levels were extremely high (18.9 g/L, range: 6.0–16.0 g/L), while IgG4 was approximately 6 times the normal limit (9.0 g/L, range: 0.08–1.4 g/L). Antinuclear antibody titer was 1:320, and his anti-SS-A antibody and antimitochondrial type 2 (M2) antibody were positive. Urinalysis showed a high level of beta-microglobulin, while proteinuria, occult blood, white blood cells and casts were within normal ranges. Other blood tests, including fecal occult blood, C-reactive protein, carbohydrate antigen, carcinoembryonic antigen, and alpha-fetoprotein, were all within normal ranges. No abnormalities were found in the complement system. The T-SPOT.TB test was negative.

**Figure 1:**
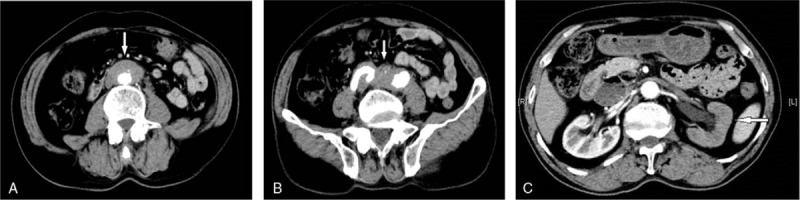
Contrast-enhanced CT of RPF before treatment. (A, B) Axial nonenhanced CT images show an irregular retroperitoneal mass (arrow) which is isoattenuating to muscle. The mass is located anterior and lateral to the lower abdominal aorta and iliac arteries. (C) Left hydronephrosis is secondary to distal encasement of the ureter by the mass. The renal excretion of contrast material was delayed (arrow). CT = computed tomography, RPF = retroperitoneal fibrosis.

Radiographs of the chest were unremarkable. Contrast-enhanced abdominal computed tomography (CT) revealed left hydronephrosis and a periaortic mass (Fig. 1). The mass surrounding the aorta appeared to be soft tissue rather than lymph nodes or tumor, suggesting left ureteral stenosis due to RPF, leading to hydronephrosis of the left kidney. Magnetic resonance cholangiopancreatography (MRCP) revealed no significant dilation of the common bile duct or the extra- and intra-hepatic bile ducts.

**Figure 2:**
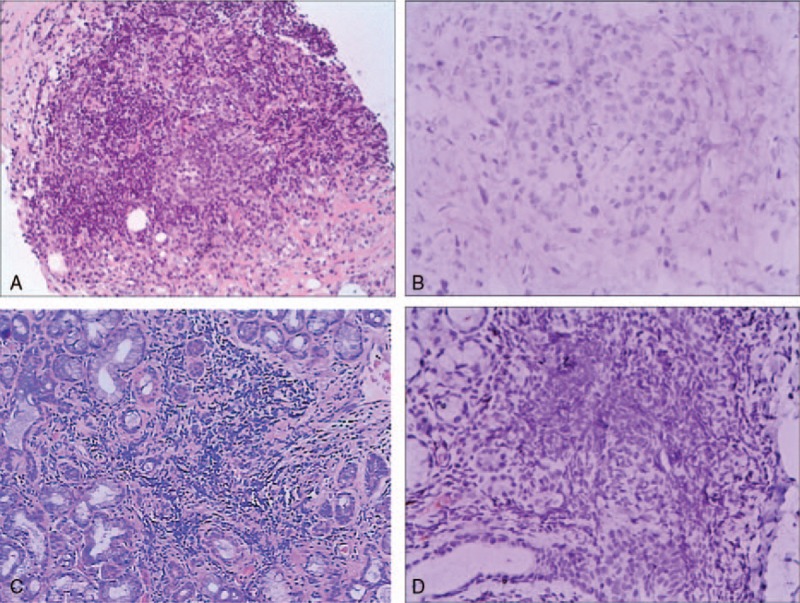
Histopathological findings of RPF and lip biopsy. (A) Hematoxylin and eosin staining of the left parotid gland demonstrates lymphoplasmacytic infiltration, fibrosis and fibrin accumulation. (B) Immunohistochemical staining of IgG4 in RPF reveals no IgG4-positive plasma cells. (C) Hematoxylin and eosin staining of lip biopsy demonstrates a decreased number of gland bubbles and heavy infiltration of lymphocytes. (D) Immunohistochemical staining of IgG4 in lip biopsy reveals no IgG4-positive plasma cells. RPF = retroperitoneal fibrosis.

The patient underwent biopsy of the retroperitoneal mass using an autobiopsy gun under CT-guidance for further diagnosis of RPF. Penetrating tissue specimens stained with hematoxylin and eosin revealed significant infiltration of lymphocytoplasma cells, fibrosis and fibrin accumulation (Fig. 2A). However, immunohistochemical staining did not show IgG4-positive plasmacytes in the retroperitoneal mass (Fig. 2B). Despite this, IgG4-related RPF was suspected, and with patient agreement we performed a less invasive lip biopsy in an attempt to obtain pathologic evidence. Although his labial salivary gland appeared normal, and the patient exhibited mild clinical features such as xerostomia and xerophthalmia, histological examination revealed a decreased number of gland bubbles and heavy infiltration of lymphocytes (100/HFP) (Fig. 2C). IgG4 staining was again negative (Fig. 2D). Together with positive expression of anti-SS-A antibody and positive Saxon and Schirmer tests, all findings fulfilled the American–European Consensus Group classification criteria for pSS.^[5]^ In addition, the patient had intrahepatic cholestasis as well as the high serum level of antimitochondrial M2, and MRCP revealed neither strictures of the lower common bile duct nor a segmental stricture. Therefore, the presence of IgG4-SC was not determined. The patient was suspected to have underlying PBC.

**Figure 3:**
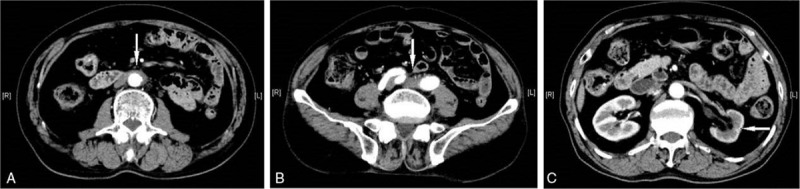
Contrast-enhanced CT of RPF after treatment. (A, B) Axial nonenhanced CT images reveal a decrease in diffuse retroperitoneal soft tissue (arrow). (C) The renal excretion of contrast material is better than that before treatment (arrow). CT = computed tomography, RPF = retroperitoneal fibrosis.

After ruling out of other possibilities, the patient was finally diagnosed with IgG4-related RPF and overlapping PBC complicated with pSS. With the patient's consent, 250 mg ursodeoxycholic acid was administered twice daily and 40 mg/d (1 mg/kg) of prednisone was administered for 30 days, which was then reduced to 30 mg/d for 15 days followed by 25 mg/d. At 45 days follow-up, the patient exhibited no signs of abdominal pain. A follow-up contrast-enhanced CT scan confirmed that the retroperitoneal soft tissue lesion was markedly reduced (Fig. 3). The ESR decreased to 29 mm/h, and IgG4 reduced to 3.18 g/L. Antinuclear antibody titer was significantly decreased to 1:100, and other laboratory data, including serum creatinine and biliary enzymes, were almost normal. The patient then received a long-term maintenance dose of 10 mg/d prednisone after steroid tapering. At six-month follow-up, the patient had no disease recurrence.

## 3 Discussion

Retroperitoneal fibrosis is an uncommon condition based on an inflammatory process, which is usually characterized by deposition of fibrotic tissue around the abdominal aorta within the retroperitoneal space. This process may extend to neighboring structures, especially the ureters and finally leading to renal failure. The onset of signs and symptoms is reported at is between 50 and 60 years, and unlike the typical features in autoimmune disease, RPF is 2 to 3 times more common likely in men than in women. The idiopathic origin of this condition accounts for approximately 2/3 of cases, while the rest are secondary to other factors such as neoplastic, infection- or drug related.^[6,7]^ According to recent reports, approximately half of RPF cases may be a symptom of relatively newly recognized, fibroinflammatory systemic condition known as IgG4-RD.^[8,9]^

IgG4-RD is a recently discovered systemic fibro-inflammatory condition characterized by the infiltration of IgG4-positive plasma cells and the formation of tumefactive lesions in the affected tissues. This disease may involve a single or multiple organs. Retroperitoneal involvement has been identified in several studies, which accounts for 20% to 56% of patients with IgG4-RD.^[9–11]^ The importance of pathological examination in the diagnosis of IgG4-RD has been increasingly recognized as other diseases mimicking IgG4-RD sometimes present with elevated serum IgG4 such as recurrent infection, systemic autoimmune conditions, and pancreatobiliary disease.^[12,13]^ The typical features of pathological change include a lymphoplasmacytic infiltrate composed of IgG4-positive plasma cells, and the combination of storiform fibrosis and obliterative phlebitis would significantly raise diagnostic specificity.^[14]^ However, the absence of pathological evidence is fairly common in clinical practice. The most common reason for this is that biopsy performance is difficult in tissues from deep organs such as the retroperitoneum, kidney, pancreas, ocular cavity, and periaorta. In the present case, we attempted CT-guided biopsy to confirm the diagnosis of IgG4-RD. However, immunohistochemical staining did not show IgG4-positive plasmacytes in the retroperitoneal mass of this patient. Possible reasons for this negative finding are as follows: It has been reported that the detection of IgG4-positive plasma cells in retroperitoneal tissue may be difficult than in other organs involvement of IgG4-RD. Consequent delay between disease onset and diagnosis lead to low specific clinical picture of RPF, hence the biopsy material usually show domination of fibrosis over cellular infiltration.^[15]^ In addition, the degree of enhancement after administration of a contrast agent, expressed in Hounsfield units (HU), depends on the activity of the inflammatory process. The present case demonstrated a relatively low level of HU compared with the nonenhanced stage (60.5 vs 51.7), which indicated a late inactive inflammatory phase.^[16]^

The lack of pathologic evidence enhanced the value of serum IgG4 in the diagnosis of IgG4-RD. As the least prevalent of the 4 IgG subclasses, IgG4 makes up to 80% of total IgG after chronic exposure to antigen. In the case of IgG4-RD, it can be rised to 50 times the upper limit.^[17]^ An elevated serum IgG4 concentration has been uesed as the cut-off value for the diagnosis of IgG4-RD in the current diagnostic criteria. According to the Comprehensive Diagnostic Criteria, the upper limit of normal (ULN) for serum IgG4 is accepted to be 135 mg/dL.^[14]^ Previous studies have reported that the sensitivity of IgG4 for the diagnosis of IgG4-RD ranged from 82.1% to 97%, whereas the specificity ranged from 79.6% to 97%.^[15,18–22]^ Moreover, recent research suggests that an elevation in serum IgG4 over 2 times the ULN contribute to distinguishing between IgG4-RD and non-IgG4-RD diagnoses, predicting multiple-organ involvement and the recurrence of the disease.^[23]^ High specificity is inevitably associated with lower sensitivity. Hence the optimal use of serum IgG4 concentrations as an initial diagnostic test, offers important support of the diagnosis of IgG4-RD if it is ≥2.8 g/L, as well as a significant argument against the diagnosis if it is normal. In the present case, the serum IgG4 concentration was approximately 6 times greater than 1.4 g/L, According to the current diagnostic criteria, a complete diagnosis of IgG4-RD including typical organ involvement, histologically compatible features and increased serum IgG4 level,^[14]^ when only typical organ involvement and elevated serum IgG4 (>135 mg/dL) are present, is defined as possible. The present case showed RPF and unilateral ureteral occlusion, with an elevated serum IgG4 despite the absence of increased IgG4-positive plasma cell infiltration. After ruling out potential diseases caused by parasitic worms, allergy or tumor, which can also lead to high levels of IgG4, we concluded that IgG4-RD was the most likely diagnosis.

Primary biliary cirrhosis is a chronic cholestatic disease with a progressive course, which is characterized by the presence of serum antimitochondrial antibodies and histological nonsuppurative destructive cholangitis. In our patient, the MRCP findings excluded the presence of IgG4-SC, and PBC was diagnosed by elevated serum anti-M2 antibody level, which has high diagnostic sensitivity and specificity for PBC. PBC, an autoimmune liver disease, is distinct from IgG4-SC, as IgG4-SC is a typical biliary manifestation of IgG4-RD, which shows segmental strictures, long strictures with prestenotic dilatation, and strictures of the lower common bile duct. To date, a few cases of overlapping PBC and idiopathic RPF have been reported.^[24,25]^ It is suggested that a small subset of PBC patients may have IgG4-related RPF. However, the clinical features of overlapping IgG4-RD and PBC are unknown due to its rarity. An underlying common pathogenesis may exist in these 2 diseases. It was reported that Toll-like receptors (TLRs) and natural killer cells play an important role in PBC patients.^[26]^ Stimulation with TLR3 and TLR4 ligands can enhance IgG4 production by peripheral blood mononuclear cells in IgG4-RD patients.^[27]^ Therefore, abnormal innate immune responses against microbial antigens may contribute to the pathogenesis of both IgG4-RD and PBC.

Except for pancreas, salivary glands have been reported to be among the most frequent organs involved in IgG4-RD (IgG4-related sialadenitis). Interestingly, IgG4-related sialadenitis shares similar clinical manifestations with an autoimmune disease, pSS, both conditions may have sicca symptoms, glandular enlargement, hypergammaglobulinemia, hypocomplementemia, and circulating antinuclear antibodies. However, they are entirely different diseases with different immunological backgrounds, research indicated that the incidence of anti-Ro and anti-La reactivity is infrequently found in patients with IgG4-related sialadenitis,^[28]^ and their salivary glands are infiltrated by a large number of IgG4+ plasma cells and IgG4-related sialadenitis symptoms respond promptly to steroids. The presence of exocrine dysfunction including positive Saxon and Schirmer tests, high serum level of immunoglobulin, positive anti-SS-A/Ro autoantibody finding and mononuclear cells infiltration in the labial salivary gland without the presence of IgG4+ plasma cells in our patient strongly suggested pSS. The coexistence of IgG4-RD and pSS has been rarely reported.^[29]^ Only one IgG4-RD case in 2594 pSS cases has been reported in the SICCA registry database.^[30]^ It is considered that the present case exhibited 2 different diseases. The identification of similar cases is required to elucidate the mechanism of IgG4-RD and pSS.

In summary, we report a case of a 69-year-old Chinese man who developed IgG-related RPF, PBC and pSS, who responded promptly to prednisone therapy. The combination of these diseases suggests that common immunogenetic factors may be involved in the development of IgG-related RPF, PBC, and pSS. A nationwide survey of the overlap between these diseases is needed to elucidate the clinical features.

## Author contributions

**Data curation:** Meng Li, Yihong Fan.

**Resources:** Xuan Huang.

**Supervision:** Yihong Fan.

**Writing – original draft:** Xuan Huang.

**Writing – review & editing:** Bin Lu, Meng Li, Lu Zhang.
